# Coalescent-Based Species Delimitation in Herbaceous Bamboos (Bambusoideae, Olyreae) from Eastern Brazil: Implications for Taxonomy and Conservation in a Group with Weak Morphological Divergence Coupled with Low Genetic Diversity

**DOI:** 10.3390/plants12010107

**Published:** 2022-12-26

**Authors:** João Paulo S. Vieira, Alessandra Selbach-Schnadelbach, Marcos Braz, Patrícia L. Ribeiro, Cássio van den Berg, Reyjane P. Oliveira

**Affiliations:** 1 Departamento de Ciências Biológicas, Universidade Estadual de Feira de Santana, Av. Transnordestina s.n., Novo Horizonte, Feira de Santana 44031-460, Bahia, Brazil; 2 Instituto de Biologia, Universidade Federal da Bahia, Av. Barão de Jeremoabo s.n., Ondina, Salvador 40150-170, Bahia, Brazil; 3 Centro de Ciências Agrárias, Ambientais e Biológicas, Universidade Federal do Recôncavo da Bahia, Cruz das Almas 44380-000, Bahia, Brazil

**Keywords:** species delimitation, BPP, *Caatinga*, Atlantic Forest, herbaceous bamboos, endangered species

## Abstract

Species delimitation in herbaceous bamboos has been complex and, in some genera, a great part of its diversity has been confirmed only based on genetic information, as is the case of the genus Raddia. It includes nine species, all occurring in Brazil, but only *R. portoi* predominates in dry forests of the Northeast associated with the *Caatinga* phytogeographic domain. This species is morphologically close to *R. angustifolia*, which is known for a single location in the Atlantic Forest in Southern Bahia, and is considered to be threatened by extinction. Besides problems with taxonomic focus, actions for its conservation are complicated because it is not certain if it must be considered an independent species or included in the more widespread *R. portoi*. In this study, we used coalescent multispecies (MSC) theory approaches combined with genetic structure analyses in an attempt to delimit these two species. Different analyses were congruent and the species delimitation using MSC inferred distinct lineages supporting their recognition as two species. These results solved the taxonomic doubts and also showed the power of these approaches to delimit species as lineages, even in groups with weak morphological divergence and low genetic variability, and also impacting our knowledge for conservation purposes.

## 1. Introduction

Species delimitation, alongside phylogeny reconstruction, are the main objectives of Systematics [1]. The task of delimiting species can be defined as the splitting of the observed diversity into intra- and inter-populational scales. Traditional molecular methods to delimit species using phylogenetic species concept (i.e., reciprocal monophyly inferred using gene trees) are prone to numerous well-known caveats [2,3,4], essentially when dealing with recently diverged species that may not yet show monophyly.

Coalescent models (Multi-Species Coalescent, MSC) allow for estimating the species of trees without the need for allele monophyly [3]. Unlike a multilocus phylogenetic inference, which is usually performed by concatenating the different genes and performing a single analysis to produce a gene tree, coalescence approaches seek to reconstruct the species tree [4]. Coalescent-based approaches are considered more appropriate for delimiting species because they better describe the stochastic nature of the speciation process [3,4]. The method implemented by Yang and Rannala [5,6] uses Bayesian species delimitation and requires the user to assign the individuals to populations and also provide a ‘guide tree’ [4].

Genetic clustering approaches are useful for species discovery without a priori information about groups and relationships among them. Two efficient and popular clustering methods are STRUCTURE [7] and Gaussian clustering [8]. Since these groupings do not access the evolutionary divergence of clusters, these would better be used as a starting point for species boundaries investigation using different methods [9]. Therefore, a possible workflow is to run a cluster analysis to find the estimate of the number of populations and assignments and after that run the Bayesian delimitation analyses [9,10]. Studies of population genetics also can reveal deep relationships among closely related populations and have been applied successfully in the delimitation of species from taxonomically problematic groups [11,12].

An interesting group to test the power of such methods is the herbaceous bamboos (the grass tribe Olyreae). This group inhabits mainly Neotropical forests and is often underestimated in most systematics studies, having a great unexplored potential for discovery of new species and identification of new lineages. Problems of species delimitation are still common in various groups of Olyreae, for example in Raddia Bertol [13,14], Piresia Swallen [15], and Eremitis Döll [16,17,18].

The genus Raddia occurs almost exclusively in forests from eastern Brazil, with exception of *R. guianensis* (Brongn.) Hitchc., which also occurs in northern Brazil and neighboring countries in northern South America [19]. The species boundaries within Raddia were recircumscribed by Oliveira and colleagues [13,14], based on both population genetics and morphometric studies, which supported the split of the complex *R. brasiliensis* into five species, four of them described as new taxa.

**Figure 1:**
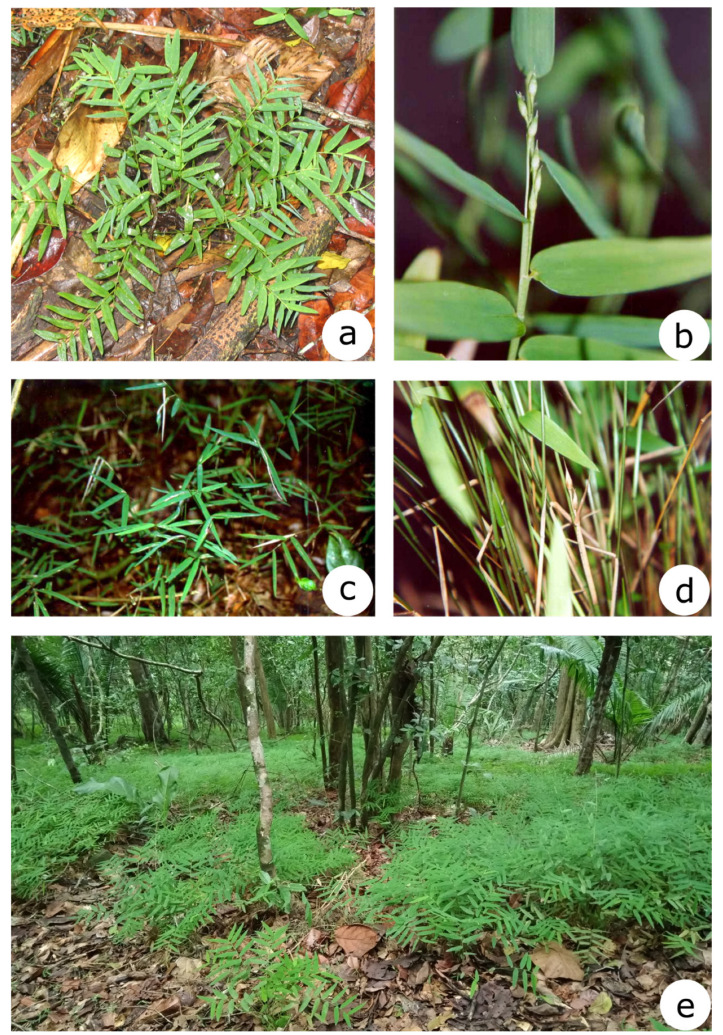
Raddia species: (**a**), *Raddia portoi*; (**b**), Inflorescence of *R. portoi*; (**c**), *R. angustifolia*; (**d**), Inflorescence of *R. angustifolia*, (**e**) Large population of *R. portoi* in the city of Cachoeira, Bahia.

Two species of this genus, *Raddia portoi* Kuhlm. and *R. angustifolia* Soderstr. & Zuloaga, are morphologically similar presenting delicate and numerous leaf blades in each culm, which are usually narrower in *R. angustifolia* [20]. This latter species has a smaller number of spikelets per female inflorescence (2-3) located in basal regions of culm, whereas *R. portoi* has 3-4(-7) spikelets per female inflorescence located at the median region of culm [14,20] (Figure 1). These characters may overlap [21], and for this reason, both species are often considered weakly distinguished [21,22]. *Raddia brasiliensis* Bertol. may also eventually be confused with *R. portoi*, as observed in misidentifications in herbaria, mainly due to the dimensions and consistency of the leaf blades [21]. Therefore, this overlap is observed only in a few individuals, because *R. brasiliensis* and its allies (the complex *Raddia brasiliensis*) frequently present leaf blades with very large dimensions when compared to both *R. portoi* and *R. angustifolia* [13,14,22].

*Raddia portoi* is the only species of the genus associated with dry forests within the Brazilian *Caatinga* phytogeographic domain, distributed from Ceará to northern Minas Gerais states [23]. Therefore, it rarely can also be found in dry environments associated with the Atlantic Forest domain in southern Bahia (R. P. Oliveira, 2006). In the latter region, we found the known populations of *R. angustifolia*, occurring only between Jussari and Itajú do Colônia municipalities [20,21].

Taxonomic decisions, considering both morphological and genetic criteria may be critical in this case, and have important implications, including their use for conservation strategies, since *Raddia angustifolia* has been indicated as critically endangered (CR) [24,25,26]. Thus, it is crucial to certify if we are dealing with one or two independent lineages, corresponding to distinct species, to concentrate conservation efforts for the preservation of *R. angustifolia* genetic diversity.

The only molecular study that included all accepted species of Raddia was the phylogeny of Oliveira et al. [19], which used rDNA and plastid molecular markers, but found

Low variation in both partitions and therefore the infra-generics relationships are not well resolved, and it was not possible to support them as separate species based on these studies. In turn, posterior studies involving this genus did not include *R. portoi* or *R. angustifolia*, but only one or a few accessions of other Raddia species, focusing on the relationships above the genus level [27,28,29].

Because populational level studies and methods not based on allele monophyly are appropriate when performing genetic analyses in groups with recent evolutionary origin or low molecular variation, as can be noticed in Raddia [19,28,30], here we apply two independent molecular approaches to species delimitation in *R. portoi* and *R. angustifolia*: (1) DNA sequence data to perform Bayesian species delimitation, and (2) ISSR genetic markers to perform populational structure analysis. We aim to understand how these very morphologically similar species relate with each other from an evolutionary perspective and if we can support them as distinctive species. We also generate data for subsidizing conservation strategies.

## 2. Results

We obtained 70 sequences for *ITS* marker, 58 from *Raddia portoi,* 9 from *R. angustifolia,* and 3 from *R. brasiliensis*; for the *trnD*-*trnT* spacer, we obtained 60 sequences, 51 from *R. portoi,* 6 from *R. angustifolia,* and 3 from *R. brasiliensis*. The final alignment was 859 bp for *ITS* sequences and 1044 bp for *trnD*-*trnT* sequences. In the BPP analyses, the use of different starting guide trees does not affect the Bayesian model selection and the results obtained were consistent across the independent runs and tested schemes combinations schemes for prior means for θs and τs. All runs reported a high posterior probability (PP) for the model with three distinct lineages (P[3] ≥ 0.99). Additionally, all three tested species receive strong support as distinct lineages (*R. portoi*, 1.0 PP; *R. angustifolia*, 0.997 PP; *R. brasiliensis,* 0.997 PP) and ((A, B), P) was the tree with the best PP support (Table 1). The 95% credibility set of trees included all three possible phylogenies. The independent runs exhibited proper mixing as indicated by the fact that independent runs produced similar results.

**Figure 2:**
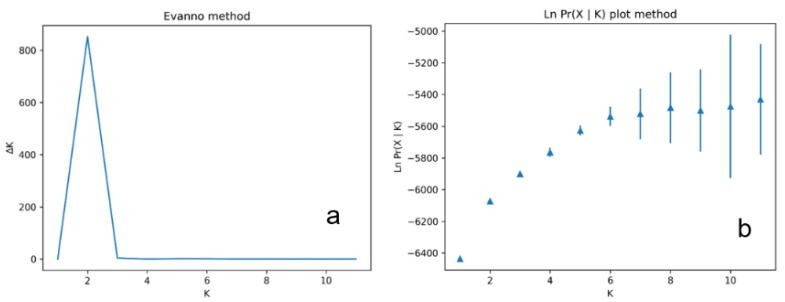
Graphs used to determine the best value for *K* based on 20 independent runs of the STRUCTURE program. (**a**), Evanno method; (**b**), Ln Pr (X | *K*) plot method, in which the vertical blue lines represent the standard deviation.

We obtained a total of 90 polymorphic ISSR loci (a mean of 11.25 loci/primer). The number of loci per population ranged from 72 in the populations PIB and PFS of *Raddia portoi,* to 81 in the population BSE of *R*. *brasiliensis*. The ∆*K* score supported the presence of only two genetic clusters from all populations (Figure 2a); however, the Ln Pr (X|*K*) plot (Figure 2b) indicated *K* = 5.

**Figure 3:**
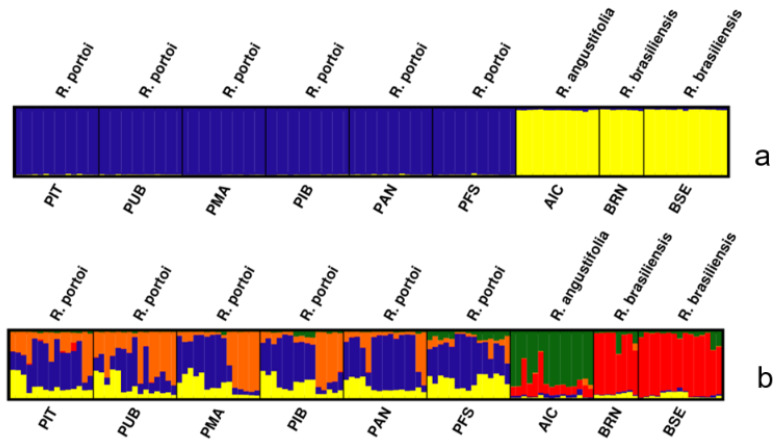
Graphic representations of the structure of the populations of Raddia created based on 20 runs of STRUCTURE with ISSR data, choosing *K* = 2 (**a**) and *K*= 5 (**b**). Different colors represent different gene pools, and each vertical bar represents an individual. For population codes, see the sampling subitem on material and methods.

For *K* = 2, the scheme of individuals from the two genetic clusters showed two well-defined clusters from all nine populations. Populations of *Raddia angustifolia* and *R. brasiliensis* consisted of one shared genetic cluster, and the populations of *R. portoi* comprise a different one. (Figure 3a). In the *K* = 5 plot, the populations of *R. brasiliensis* and *R. angustifolia* present two different gene pools with little admixture, and the *R. portoi* populations are almost exclusively constituted of three other pools (Figure 3b).

**Figure 4:**
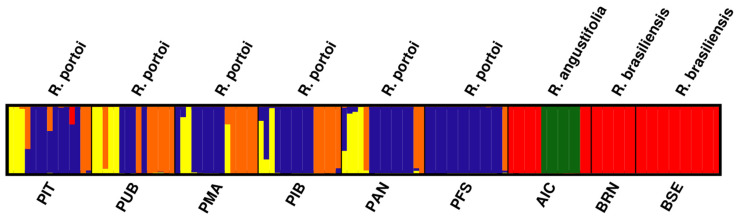
Graphic representations of the structure of the populations of Raddia created using the Gaussian cluster analysis on ISSR data, showing the proportion of gene pools in each population, with different colors representing different gene pools. Colors are consistent with Figure 3b.

The PCO modal clustering analysis revealed the *K* = 5 model as the best based on the BIC score. These results are similar to the *K* = 5 result of STRUCTURE, regarding both the number and distribution of the gene pools (Figure 4).

The AMOVA from all populations resulted in Φ_PT_ = 0.20, equally divided into population divergence and region divergence (*p* = 0.001 for both), with 80% of the total variation within populations (Table 2).

The Φ_PT_ values of pairwise populations were significant in almost all comparisons, except for PIB x PIT (*p* = 0.09), and those varied between 0.04 and 0.28 (Table 3).

**Figure 5:**
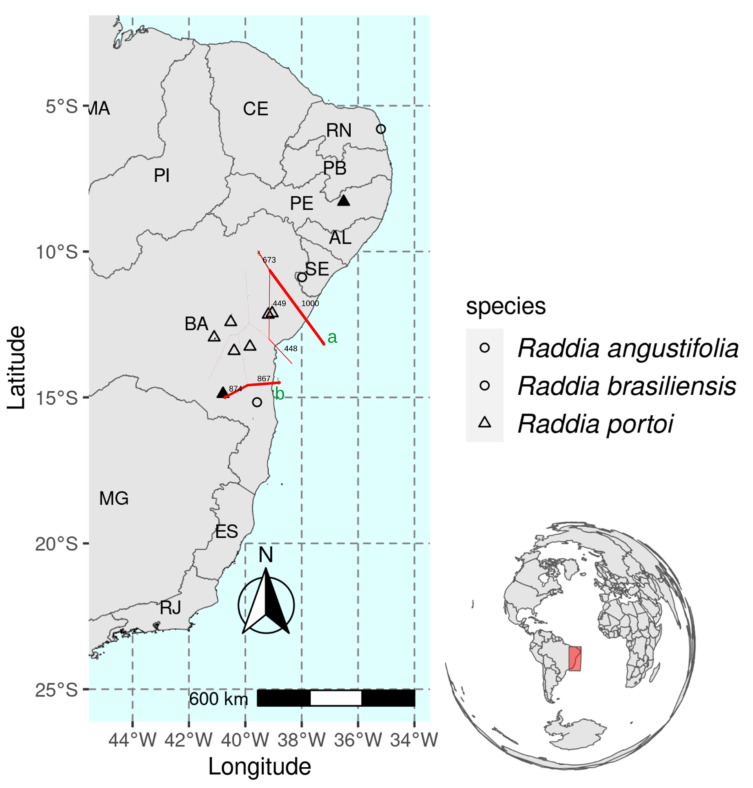
Population locations and inferred genetic barriers to gene flow. The barriers **a** and **b** were inferred from the real data (90 polymorphic ISSR loci). Values above barriers represent the number of inferred barriers and the thickness of the barriers is proportional to their support, generated via 1000 bootstrap replications of the empirical data. Full triangles are *Raddia portoi* populations sampled only for sequence data.

Analysis of genetic and geographic distances highlighted a highly supported (100% bootstrap) barrier isolating populations of *R. brasiliensis* (Natal—RN and Tobias Barreto—SE) from all other populations. A second barrier (87% bootstrap) suggested the isolation of *R*. *angustifolia* from populations of the other two species (Figure 5).

## 3. Materials and Methods

### 3.1. Sampling

We sampled the only known population of *Raddia angustifolia* and eight populations of *R. portoi* in Bahia, Sergipe, Rio Grande do Norte, and the Pernambuco states in Northeast Brazil, including the type localities of both *R. portoi* (Itaetê, Bahia) and *R. angustifolia* (Itaju do Colônia, Bahia) (Figure 1). We also included two populations *of R. brasiliensis* Bertol. for comparison purposes. We collected young leaves taking care to avoid collecting clonal individuals. Vouchers were deposited in the herbarium of the Universidade Estadual de Feira de Santana (HUEFS), except for population from Tobias Barreto, deposited at the herbarium of the Universidade Federal de Sergipe (ASE; acronym by Thiers [31]) (Table 4).

### 3.2. DNA Isolation, PCR Amplification, and Sequencing

We used 20 µg of silica gel dehydrated leaves using the CTAB 2× protocol described by J.J Doyle and J. L. Doyle [32], scaled to microtubes. After quantification in agarose, we amplified the DNA fragments by PCR using TopTaq Master Mix Kit ^®^ (Qiagen Corp.). The amplification protocol suggested by the manufacturer was adapted to a total volume of 15 μL, containing 0.2 μM of each primer and 10–20 ng template DNA.

The ribosomal DNA *ITS* region was amplified with the *17SE* and *26SE* primers [33]. PCR was performed with an initial denaturation step at 94 °C for 3 min, followed by 28 cycles of 1 min at 94 °C, 1 min at 50 °C, and 3 min at 72 °C, with a final 7 min extension at 72 °C.

The plastid spacer *trnD*-*trnT* was amplified using the universal *trnD* primer [34] and a *trnT* specific primer for Poaceae, due to an inversion that species of this family present at the annealing site of primers [35]. PCR was performed with an initial denaturation step at 94 °C for 1 min, followed by 30 cycles of 30 s at 94 °C, 40 s at 52 °C, and 70 s at 72 °C, with a final 5 min extension at 72 °C.

PCR products were purified by precipitation using PEG (polyethylene glycol) [36] and were sequenced using Big Dye Terminator ^®^ v. 3.1 kits with the same primers used in PCR for *trnD*-*trnT.* For *ITS* we used *ITS4* [37] and *ITS92* [38] primers. Products were run in an ABI 3130XL Genetic Analyzer ^®^ (Applied Biosystems, Foster City, CA, USA) at LAMOL/UEFS.

### 3.3. Sequence Alignment and Bayesian Species Delimitation

Electropherograms were edited in Staden Package 2.0.0b11 [39] saving the consensus sequence. Consensus sequences were deposited in GenBank (Table 4). The matrices were constructed by aligning these sequences with MAFFT [40] algorithm using 100 bootstrap repeats at the GUIDANCE2 server [41]. 

We used Bayesian Phylogenetics and Phylogeography (BPP) v4.3 [42] to reconstruct the phylogeny and model the speciation of the taxa. BPP uses a coalescent approach and is useful for delimiting species using molecular data without relying on the criterion of reciprocal monophyly and therefore avoiding problems with recently diverged lineages and incomplete lineage sorting [4]. Initially, we ran an A00 analysis to infer the parameters θ (theta, effective population size) and τ (tau, divergence time) [43], using the phylogeny ((A, B), P) (A, *Raddia angustifolia*; B, *R. brasiliensis*; P, *R. portoi*) based on the genetic structure results (see Results), resulting in the means 0.0001 for θs and 0.0015 for τ_ABP_.

After that, we run an A11 analysis (species delimitation and species tree estimation). This analysis was implemented on version 3 of the program and, differently from an A10 analysis, eliminates the need for a user-specified guide tree [6,43]. As the posterior probabilities calculations of the delimitations are not based on a fixed phylogeny, phylogenetic uncertainty is accounted for in the calculation of the posterior probabilities of delimitations.

For the A11 analysis, inverse-gamma priors based on A00 run results were diffusely informed for θ ~ IG(3, 0.0002) and τ ~ IG(3, 0.003). As has been noted that those priors can impact the posterior probabilities, we tested the effects of different priors on the obtained results using nine schemes similar to what was done by Leaché and Fujita [10]: sometimes using an effective population size larger than estimated, sometimes smaller, allowing the parameters mean to vary over two degrees of magnitude [43]; the same was done for the divergence time parameter (Table 1). We also tested the effect of different starting guide trees on the results, using all three possible phylogenies for the taxa as starting guide trees. These should lead to the same results with algorithm convergence [43].

Each run consisted of a burn-in period of 8000 iterations and a sampling period of 100,000 iterations, logged every two, for a total of 50,000 samples. The fine-tune parameters were obtained by trial and error as recommended in the BPP manual and can be checked on the control files (File S1). Two independent runs were carried out in each case to ensure the correct mix of the Markov chains. 

### 3.4. ISSR-PCR Amplification and Polymorphism Test

Fragments were amplified by PCR (Polymerase Chain Reaction) using 1× TopTaq Master Mix ^®^ (Qiagen Inc., Hilden, Germany), 1× CoralLoad Concentrate, 4.8 pmol of primer, and 10 ng template DNA to a final volume of 7.2 μL. We tested the 20 primers described on the ISSR Resources website, archived at https://web.archive.org/web/20170605202905/http://www.biosci.ohio-state.edu/~awolfe/ISSR/protocols.ISSR.html (accessed on 25 July 2020).) and from the UBC primer kit #9 (Biotechnology Laboratory, University of British Columbia, Canada) with one sample of each population, chosen randomly, for the verification of the amplification profile. The primers MANNY: (CAC)_4_ -RC, MAO: (CTC)_4_-RC, ISSR-4: (GA)_8_-YC; M9: (GACAC)_3_; DAT: (GA)_7_-RG; TERRY: (GTG)_4_-RC; 898: (CA)_6_-RY and 901: (GT)_6_-YR presented satisfactory resolution and polymorphism and were selected for genotyping. To certify the reproducibility of the obtained band patterns, we performed replications of the reactions for 25% of the total sample size, using samples chosen at random.

The amplifications were based on Wolfe et al. [44] as follows: initial denaturation at 94 °C for 90 s, followed by 35 cycles of 94 °C for 40 s, 45 °C for 45 s, 72 °C for 90 s, followed by two more amplification cycles (94 °C for 45 s, 44 °C for 45 s, 72 °C for 90 s), and a final extension of 72 °C for 7 min.

Amplicons were subjected to electrophoresis in a 2% agarose gel with SB buffer, under 100 V and 80 mA for 120 min with the marker Ladder 100 PB (Ludwig Biotecnologia, Alvorada, Brazil) to determine the size and concentration of the obtained fragments, followed by photo-documentation. These data were entered into the GelCompar II ^®^ v. 5.1 (Applied Maths NV, Sint-Martens-Latem, Belgium) in order to assess size homology and produce a binary matrix with zeroes representing absences, ones for the presences of bands, and −1 for missing data (File S2).

### 3.5. Species Delimitation Using Cluster Algorithms

For the ISSR matrix, we used the model-based Bayesian approach implemented in STRUCTURE 2.3.4 [7,45,46] to infer genetic clusters. All analyses to estimate allele frequencies of populations assumed that an individual is not restricted to one single population. Accordingly, we tested the assignment of individuals into one to 11 genetic clusters (*K* = 1—11) using the admixture model with correlated allele frequencies. The analysis of each cluster consisted of 20 independent runs of 300,000 Markov chain Monte Carlo (MCMC) replicates following an initial burn-in of 100,000. To estimate the number of clusters, we used the ∆K [47] and the Ln Pr (X|K) plot methods [48], both calculated with the Structure Harvester vA.2 program [49]. We used the library Matplotlib [50] to create the graphs for visualization. We used the program CLUMPP v. 1.1.2 [51] with the full search method and G′ statistics to group the independent replicates into only one array. We used the program Distruct 1.1 [52] to draw the graphs for the matrices produced by CLUMPP and mclust.

We conducted an analysis of principal coordinates modal clustering (PCO-MC) [53,54] within the R environment [55]. First, we run a PCoA analysis [56] using the ape 5.4–1 package [57]. We used Jaccard similarity indexes [58] obtained from the ISSR data matrix using the vegan 2.5–6 package [59]. We analyzed the scores of the PCO’s from the first nine axes (42.7% of the variation) to estimate the number of genetic groups and the classification of each population within these groups using model-based clustering through the mclust 5.4.6 package [60] whereby we retrieved the number and the classification of every individual in each inferred cluster. The best model was selected based on the Bayesian information criterion (BIC). The graph representing the clusters was also drawn using Distruct.

We calculated the distribution of genetic variation through an Analysis of Molecular Variance (AMOVA) [61] in GenAlEx 6.5 [62]. We also assessed regional genetic structure (*Caatinga* and Atlantic Forest). To verify if the result is not only a sampling artifact, we contrasted the empirical values with a null distribution generated by 999 randomizations of the original data, in which the individuals were swapped between populations by chance.

Finally, we tested for genetic barriers among populations using an autocorrelation analysis between genetic and geographic distances using Barrier v. 2.2 [63]. For this, we used a Nei genetic distance [64] matrix, and the two more likely barriers were drawn. The significance level of the barrier was evaluated by 1000 bootstrap replications of the original data matrix. The matrices were calculated using AFLP-SURV 1.0 [65], and the method described by Zhivotovsky [66] with a uniform prior to inferring allelic frequencies.

## 4. Discussion

As previous taxonomic studies involving the genus Raddia have demonstrated a weak morphological separation between *R. portoi* and *R. angustifolia*, we used here different species delimitation approaches, even though both are from the same source of variation and DNA data, it sums up the previous information and gives greater reliability in the delimitation of these species. The approaches used here are generally used to find structure among populations of the same species, but they can also be used to find cryptic lineages within species complexes and taxonomic species in which it is speculated that they may contain more than one evolutionary lineage (e.g., [9,67,68,69,70]).

Several species delimitation approaches using species trees are available, but most of them fail to accommodate uncertainties involved in the process, such as incomplete lineage sorting and sampling and phylogenetic errors in the gene tree reconstruction [4]. The Bayesian species delimitation used by the program BPP is able to accommodate all of these and has been reported to outperform different approaches [68,71,72,73]. In the present work, the result of the Bayesian species delimitation analysis using BPP was concordant with what was found in the structure analyses, indicating that all three putative species can be considered different lineages. The model with three lineages was the one with the highest posterior probability in all BPP runs (Table 1). Besides, using a threshold of 95–99% of posterior probabilities [43] all three taxonomic species had a high probability of consisting of evolutionary lineages.

In the STRUCTURE analysis, we chose to test two approaches to choose the best number of *K* groups, since the ∆*K* method is biased towards the selection of *K* = 2 [74]. Here, *K* = 2 was also the value indicated by this method. Looking at the Ln Pr (X|*K*) graph, we see that it reaches a plateau at *K* = 5. We decided to present the two graphical representations. Despite having a different number of clusters, both structure graphs indicate a similar pattern, with the populations of *Raddia portoi* showing up separate from the others, and with sharing of gene pool (s) between the populations of *R. angustifolia* and *R. brasiliensis*. The five clusters found in the mclust analysis also indicate the separation between the populations of *R. portoi* from the others since three of the clusters (represented by the colors yellow, orange, and blue) are almost exclusive to the populations of this species; one is exclusive to the population of *R. angustifolia,* and the rest are shared by the populations of *R. brasiliensis* and the population of *R. angustifolia*.

Based on the high Φ_ST_ value found in AMOVA, the structure found here most likely reflects speciation processes rather than intraspecific population structure. The Φ_ST_, an F_ST_ analogue, of 0.20 indicates that there is less than one migrant per generation between the populations (*N_e_m* < 1), which is considered a rule of thumb to assume populations different enough to be treated as evolutionarily significant units (ESU) [75,76] sensu Moritz [77], what means that we are dealing with distinct lineages instead of a highly polymorphic one. The pairwise Φ_ST_ results show that the greatest genetic divergences are between the *Raddia brasiliensis* populations and the others, with the majority of the Φ_ST_ values > 0.20. Among the populations of *R. portoi* this divergence was mostly Φ_ST_ < 0.10, except for the PFS population, which presents high divergences to the others of the same species. This implies an efficient gene flow between populations of the same species, with PFS being the most isolated population of *R. portoi*.

We also found a high and significant regional structure through the AMOVA analysis (Φ_RT_). The regions we tested correspond to the biomes in which the populations occur: *Caatinga* (for most of *Raddia portoi* populations) and Atlantic Forest (for *R. angustifolia* and *R. brasiliensis* populations). This result indicates that the populations of *R. angustifolia* and *R. brasiliensis*, represent distinct lineages apart from *R. portoi*, and also agrees with the fact that there is sympatry in some Raddia species [19,21].

The analysis of genetic barriers is also congruent with this result, showing that the barriers occur between putative species, and are not related to geographical distance or barriers to gene flow. For example, even though the two populations of *Raddia brasiliensis* are more than 500 km apart from each other and also separated by the São Francisco River basin, a recognized important barrier to the gene flow [78,79,80,81,82,83,84], the analysis identified no genetic barriers between them. Conversely, the population of *R. angustifolia*, despite being geographically closer to the PUB and PMA populations of *R. portoi*, is isolated from these by a highly supported genetic barrier (87% bootstrap).

### Species Boundaries in Raddia portoi and R. angustifolia and Implications for Their Conservation

Brazilian forests harbor the highest diversity of herbaceous bamboos, ca. 92 out of 127 known species, distributed mainly along the Atlantic Forest and Amazon [85]. However, their diversity can be considered underestimated due to the low number of published studies and the increasing number of newly described species and genera [15,17,18,30,86].

Bahia state contains the highest number of genera and species of Olyreae in Brazil, based on herbarium revisions and field collections [87]. It also includes the highest endemism rates in the Atlantic Forests remnants of southern Bahia. This agrees with the findings of Soderstrom et al. [88] that indicated Bahia as the highest center of diversity for American bamboos.

Herbaceous bamboos deserve special attention because they are important components of neotropical flora, and in recent decades have become extremely rare due to the intense increase in anthropic action in the Brazilian Atlantic Rainforest [89]. This is due to a myriad of factors: the herbaceous vegetation is the first to be removed during deforestation; most of the Olyreae species are comprised of small populations; they are also sensitive to forest fragmentation and cover loss due to their intolerance to direct insolation [21,87].

Previous phylogenetic studies including Raddia were inconclusive regarding the relationships and delimitation of *R*. *portoi* and *R*. *angustifolia* [19]. Nonetheless, our results here are congruent across all analyses, indicating that *R*. *angustifolia* and *R*. *portoi* are different lineages. Genetic similarity between populations of *R*. *portoi* is higher than that between *R*. *angustifolia* and populations of *R*. *portoi*. Additionally, the population of *R. angustifolia* showed more genetic similarity with the populations of *R. brasiliensis* than with *R. portoi* itself in the STRUCTURE results and the BPP species trees. Although, this is a limited phylogeny since the 95% credibility set includes all three models. This low phylogenetic information has been observed in other studies with the A11 analysis [43]. The high morphological similarity between these species and the lack of phylogenetic resolution can be an indication of recent divergence for this group, as pointed out by Ruiz-Sanchez et al. [28], which indicated less than 1 ma for the Raddia crown group.

According to our results, we can confirm that *Raddia angustifolia* represents a different lineage from *R. portoi*, reaffirming its conservation status as a highly endangered species, for which conservation measures should be directed in order to preserve its genetic diversity. Populations of *R. angustifolia* are located in highly disturbed areas, so we should prioritize its conservation. It is considered endemic to southern Bahia, occurring on disturbed fragments of the Atlantic Forest [20,24], the most devastated and threatened ecosystem of Brazil [90]. *Raddia angustifolia* was also recently listed in the Brazilian threatened flora compilation [25].

Regarding *Raddia portoi*, it has a wider distribution in dry forests of eastern Brazil, coinciding with the limits of the Brazilian semiarid region, and has not been listed as threatened. Although its populations are more numerous, we cannot ignore the fact that the populations are distributed in areas highly fragmented by human activities and the fact that the *Caatinga* is the less protected phytogeographic domain of Brazil [91].

## Figures and Tables

**Table 1:** Prior schemes tested in BPP. Priors for scheme 1 were estimated from the data and the other schemes were used to test the effects of different priors and were set to allow the parameters means to vary over two degrees of magnitude. Additionally, the best models for the species tree and the number of species are displayed with their respective posterior probabilities (PP) values.

Scheme	Gamma Distribution for Prior	Best Species Tree and PP	Best Model for Species Number
1	θ ~ IG(3, 0.0002) and τ ~ IG(3, 0.0003)	((A, B), P) 0.61	P[3] = 0.99
2	θ ~ IG(3, 0.0002) and τ ~ IG(3, 0.003)	((A, B), P) 0.61	P[3] = 1
3	θ ~ IG(3, 0.0002) and τ ~ IG(3, 0.03)	((A, B), P) 0.66	P[3] = 1
4	θ ~ IG(3, 0.002) and τ ~ IG(3, 0.003)	((A, B), P) 0.54	P[3] = 0.99
5	θ ~ IG(3, 0.002) and τ ~ IG(3, 0.0003)	((A, B), P) 0.48	P[3] = 0.99
6	θ ~ IG(3, 0.002) and τ ~ IG(3, 0.03)	((A, B), P) 0.65	P[3] = 0.99
7	θ ~ IG(3, 0.00002) and τ ~ IG(3, 0.003)	((A, B), P) 0.59	P[3] = 1
8	θ ~ IG(3, 0.00002) and τ ~ IG(3, 0.0003)	((A, B), P) 0.6	P[3] = 1
9	θ ~ IG(3, 0.00002) and τ ~ IG(3, 0.003)	((A, B), P) 0.64	P[3] = 1

**Table 2:** Analysis of molecular variance (AMOVA) for Raddia populations.

Source of Variation	Degrees of Freedom	Sum of Squares	Variance Component	Variance (%)
Among Regions	1	160.902	2.136	10%
Among Pops	7	331.091	2.133	10%
Within Pops	119	2020.992	16.983	80%
Total	127	2512.984	21.252	100%

**Table 3:** Pairwise Φ_ST_ between Raddia populations below the diagonal and *p*-values above the diagonal. For population codes see the sampling subitem on Section 3.

PIT	PUB	PMA	PIB	PAN	PFS	AIC	BRN	BSE	
0.000	0.001	0.020	0.095	0.001	0.001	0.001	0.001	0.001	**PIT**
0.082	0.000	0.001	0.001	0.001	0.001	0.001	0.001	0.001	**PUB**
0.043	0.079	0.000	0.013	0.002	0.001	0.001	0.001	0.001	**PMA**
0.018	0.070	0.049	0.000	0.001	0.001	0.001	0.001	0.001	**PIB**
0.069	0.069	0.074	0.073	0.000	0.001	0.001	0.001	0.001	**PAN**
0.121	0.179	0.167	0.095	0.149	0.000	0.001	0.001	0.001	**PFS**
0.183	0.153	0.201	0.173	0.151	0.224	0.000	0.001	0.001	**AIC**
0.191	0.205	0.263	0.236	0.217	0.278	0.204	0.000	0.002	**BRN**
0.197	0.212	0.266	0.209	0.190	0.286	0.180	0.104	0.000	**BSE**

**Table 4:** Codes, localization, vouchers, data utilized, and accession numbers for Raddia populations in this study.

Taxon	*Voucher*	Location	Code	Coordinates	Sample Size	GenBank Accession Numbers
*Raddia angustifolia* Soderstr. & Zuloaga	RPO 1077	Itaju do Colônia—BA	AIC	15°08′33″ S 39°43′28″ W	ISSR: 15; *ITS*: 8; *trnD*-*trnT*: 6	*ITS*: OP919392-OP919400; *trnD-trnT*: OP946258-OP946263
*R. portoi* Kuhlm	RPO 1450	Itaeté—BA	PIT	12°56′40″ S 41°6′20″ W	ISSR: 15; *ITS*: 6; *trnD*-*trnT*: 6	*ITS*: OP919419-OP919424; *trnD-trnT*: OP946281-OP946286
	RPO 1501	Ubaíra—BA	PUB	13°15′34″ S 39°50′9″ W	ISSR: 15; *ITS*: 10; *trnD*-*trnT*: 8	*ITS*: OP919452-OP919461; *trnD-trnT*: OP946306-OP946313
	MCD 72	Maracás—BA	PMA	13°24′24″ S 40°23′52″ W	ISSR: 15; *ITS*: 11; *trnD*-*trnT*: 7	*ITS*: OP919425-OP919436 *trnD-trnT*: OP946287-OP946293
	RPO 1439	Itaberaba—BA	PIB	12°18′11.0″ S 40°31′13.0″ W	ISSR: 15; *ITS*: 4; *trnD*-*trnT*: 5	*ITS*: OP919414-OP919418; *trnD-trnT*: OP946276-OP946280
	RPO 1548	Anguera—BA	PAN	12°9′49″ S 39°11′12″ W	ISSR: 15; *ITS*: 7; *trnD*-*trnT*: 6	*ITS*: OP919407-OP919413; *trnD-trnT*: OP946270-OP946275
	CS 314	Feira de Santana—BA	PFS	12° 6″ S 39° 2″ W	ISSR: 15; *ITS*: 6; *trnD*-*trnT*: 6	*ITS*: OP919401-OP919406 *trnD-trnT*: OP946264-OP946269
	RPO 2143	Vitória da Conquista	-	14°52′46.0″ S 40°47′28.0″ W	*ITS*: 3; *trnD*-*trnT*: 4	*ITS*: OP919462-OP919464 *trnD-trnT*: OP946314- OP946317
	MCD 126	Sanharó—PE	-	8°17′43.0″ S 36°30′46.0″ W	*ITS*: 12; *trnD*-*trnT*: 50	*ITS*: OP919440-OP919451 *trnD-trnT*: OP946296-946305
*R. brasiliensis* Bertol	DC 2674	Natal—RN	BRN	5° 47′40″ S 35° 12′39″ W	ISSR: 8; *ITS*: 3; *trnD*-*trnT*: 2	*ITS*: OP919437-OP919439 *trnD-trnT*: OP946294-OP946295
	A.P. Prata 2633	Tobias Barreto—SE	BSE	10°52′52″ S 39°59′11″ W	ISSR: 15	

## Data Availability

All relevant data are within the paper; sequence data were deposited in GenBank (accession numbers in Table 4) and the binary matrix data and BPP control files are in the supplementary materials.

## Supplementary Materials

The following are available online at https://www.mdpi.com/article/10.3390/plants12010107/s1, Supplementary file S1: BPP control files. Supplementary file S2: ISSR binary matrix.
